# 2164. Resistome patterns associated with vancomycin non-susceptibility in clinical *Clostridioides difficile* isolates

**DOI:** 10.1093/ofid/ofad500.1787

**Published:** 2023-11-27

**Authors:** Taryn A Eubank, Anne J Gonzales-Luna, Blake M Hanson, Todd Treangen, Ashlee Earl, Kevin W Garey

**Affiliations:** University of Houston College of Pharmacy, Houston, Texas; Department of Pharmacy Practice and Translational Research, University of Houston College of Pharmacy, Houston, Texas, USA, Houston, TX; The University of Texas Health Science Center, Houston, Texas; Rice University, Houston, Texas; Broad Institute of MIT and Harvard, Cambridge, Massachusetts; University of Houston, Houston, TX

## Abstract

**Background:**

*Clostridioides difficile* exhibits phenotypic resistance to multiple classes of antibiotics. Associated antibiotic resistance genes (ARGs) are commonly transferred via mobile genetic elements, which often carry distinct ARG groupings. Increases in vancomycin non-susceptible *C. difficile* have been reported and understanding associated trends in ARG co-carriage are critical for strain transmission dynamics. Here we aimed to determine *C. difficile* resistome patterns associated with vancomycin non-susceptibility (NS).

**Methods:**

Whole genome sequencing (WGS) was performed (Illumina NextSeq 500) on *C. difficile* isolated from adult patients hospitalized with *C. difficile* infection in two health systems (14 hospitals) in the Houston Area between 2016–18. Isolates were ribotyped by fluorescent PCR and susceptibility tested by agar dilution in accordance with CLSI standards. Vancomycin NS was defined by minimum inhibitory concentrations (MICs) > 2 mg/L. Genome assembly and analysis was completed through The Bacterial and Viral Bioinformatics Resource Center (BV-BRC) web resources.

**Results:**

Overall, 36% (13/36) of the cohort was vancomycin NS. The most common ribotypes were F106 (n=13), F027 (n=10), and F014-020 (n=7). Median ARG abundance was 46 (IQR 45 – 49) and included genes encoding targeted and non-specific (i.e., encoding ABC transporters or efflux pumps) resistance. Specific antibiotics/classes targeted by identified ARGs included aminoglycosides, daptomycin, diaminopyrimidines, fluoroquinolones, fosfomycin, glycopeptides, isoniazid, lincosamides, macrolides, nitroimidazoles, oxazolidinones, rifamycins, streptogramins, and sulfonamides. Median ARG abundance did not differ between vancomycin NS (46.5 genes) and susceptible (46 genes) isolates and no differences in ARG patterns were observed. ARG abundance and patterns were similar amongst ribotypes.

**Conclusion:**

*C. difficile* vancomycin non-susceptibility did not correlate with increased ARG abundance or any distinct ARG patterns. Future studies investigating interactions and implications of the *C. difficile* resistome on the microbiome and host are warranted.

**Disclosures:**

**Anne J. Gonzales-Luna, PharmD, BCIDP**, Cidara Therapeutics: Grant/Research Support|Ferring Pharmaceuticals: Personal Fees|Paratek Pharmaceuticals: Grant/Research Support|Seres Therapeutics: Grant/Research Support **Kevin W. Garey, PharmD, MS**, Acurx: Grant/Research Support|Ferring: Advisor/Consultant|Paratek: Grant/Research Support

